# Clinical Characteristics and Outcome of Jamestown Canyon Virus Infection in Hospitalized Wisconsin Patients

**DOI:** 10.1093/ofid/ofaf360

**Published:** 2025-07-29

**Authors:** Igor Dumic, Charles W Nordstrom, Janki Patel, F N U Shweta, Larry Lutwick

**Affiliations:** Department of Hospital Medicine, Mayo Clinic Health System, Eau Claire, Wisconsin, USA; Department of Hospital Medicine, Mayo Clinic Health System, Eau Claire, Wisconsin, USA; Mayo Clinic Health System, Eau Claire, Wisconsin, USA; Department of Infectious Disease, Mayo Clinic Health System, Eau Claire, Wisconsin, USA; Department of Medicine, Mayo Clinic College of Medicine, Rochester, Minnesota, USA; Program for Monitoring Emerging Infectious, Boston, Massachusetts, USA

**Keywords:** Jamestown Canyon virus, meningitis, encephalitis, emerging infection, vector-borne disease

## Abstract

**Background:**

Jamestown Canyon virus (JCV) is an emerging arboviral pathogen transmitted by mosquitoes in the United States and Canada. It is associated with neuroinvasive diseases and a wide spectrum of clinical presentations.

**Methods:**

A retrospective chart review was conducted at the Mayo Clinic Health System in Northwest Wisconsin from January 2018 to December 2024, analyzing data from 12 hospitalized patients diagnosed with JCV infection. This study aimed to characterize the clinical, laboratory, imaging, and outcome profiles of the disease. The cohort included individuals aged 19 to 75 years, predominantly male (75%), with all patients reporting outdoor activity and 75% being pet owners.

**Results:**

Only 27% of the patients recalled a mosquito bite. Among the cohort, 2 patients acquired an infection during the first trimester of pregnancy and subsequently delivered healthy infants. Coinfections with *Borrelia burgdorferi* were found in 33% of cases, while 16.7% of cases were seropositive to La Crosse virus. Magnetic resonance imaging of the brain was obtained in 5 patients and was nonspecific. Cerebrospinal fluid analysis demonstrated lymphocytic or monocytic pleocytosis in all cases where the test was performed.

**Conclusions:**

All patients experienced clinical improvement, with only 1 developing lingering neurological symptoms in the form of brain fog. This study highlights the clinical heterogeneity of JCV infections and underscores the importance of including JCV in the differential diagnosis for patients residing in or traveling from endemic areas.

Jamestown Canyon virus (JCV), an enveloped RNA virus within the California serogroup of the genus *Orthobunyavirus*, was first isolated in 1961 from *Culiseta inornata* mosquitoes in Colorado [1–5]. JCV is an emerging arboviral pathogen primarily transmitted to humans through the bite of infected mosquitoes, and >50% of reported cases in the United States originate from the Midwestern states of Wisconsin and Minnesota. Most human infections are asymptomatic or present with mild, nonspecific symptoms, resulting in significant underreporting. JCV can in rare cases cause life-threatening neuroinvasive disease. The incidence of infections has notably increased over the past 2 decades [5]. Approximately 50% of reported cases involve neuro-invasion [1–10], with symptomatic infections typically presenting as fever, fatigue, headache, encephalitis, or meningitis [1–5]. Diagnosis is based on clinical presentation, patient history, and epidemiologic context and is confirmed through acute and convalescent serological testing of serum or cerebrospinal fluid (CSF) [1–5]. Detailed clinical data on JCV remain limited, with most insights derived from case reports and small case series.

## METHODS

We conducted a retrospective chart review of patients admitted to the Mayo Clinic Health System in Northwest Wisconsin between January 2018 and December 2023 who tested positive for JCV infection. The study was classified as minimal risk, exempting it from further review given its retrospective nature. Diagnosis of JCV was confirmed through serologic testing, specifically a positive JCV-specific immunoglobulin M (IgM) result in either serum or CSF. Given the significant cross-reactivity of antibodies within the California serogroup, positive IgM results were further validated by a plaque reduction neutralization test (PRNT) conducted at the Centers for Disease Control and Prevention (CDC) through the Wisconsin State Laboratory.

Coinfection with *Borrelia burgdorferi* was considered if the patient had a positive polymerase chain reaction–based test for Lyme disease or demonstrated seropositivity along with clinical characteristics diagnostic of probable or possible Lyme disease (eg, a typical rash). Patients with only positive antibody results but no signs or symptoms of disease were classified as having seropositivity without coinfection.

Demographic information including the month of diagnosis, pet ownership, history of insect bites, and outdoor activities was extracted from the patients' medical records. Additionally, we collected detailed information on clinical symptoms, laboratory findings, imaging results, presence of coinfections, treatments administered, and clinical outcomes.

## RESULTS

All 12 patients were diagnosed between May and October, and 75% of the patients were male, with a median age of 51 years, consistent with findings from prior case series. All patients reported engaging in outdoor activities. Only 1 patient had a remote history of prostate cancer but was not on immunosuppressive therapy; the remaining patients had no history of immunosuppression. The most common clinical symptoms were constitutional (malaise, fatigue, anorexia) and were observed in all patients, followed by fever and headache in 9 of 12 patients (75%). Encephalopathy occurred in 5 of 12 patients (41.7%), with 2 presenting with seizures and 1 with syncope. The average time from symptom onset to JCV diagnosis was 26 days, with a range of 15 to 45 days. Lumbar puncture was performed in 9 of 12 patients (75%), and CSF analysis revealed lymphocytic pleocytosis in all but 1 case, which showed monocytosis. Coinfections with *Borrelia burgdorferi* were identified in 4 of 12 patients (33.3%) based on seropositivity (IgM antibody positive), history of tick bite, and typical erythematous rash in all 4 cases. Antibodies against La Crosse virus were positive in 2 of 12 patients (16.7%), and 1 patient had multiple coinfections, including histoplasmosis and a nontuberculous mycobacterial infection. None of the patients received corticosteroids, intravenous immunoglobulins (IVIGs), or any other immunosuppressive therapy, but 10 of 12 (75%) received empiric antibacterials.

The detailed clinical characteristics, laboratory findings, and imaging results of the patients are summarized in Table 1. Brain magnetic resonance imaging (MRI) was performed in 5 of 12 patients (41.7%) and yielded nonspecific results in all cases. Lumbar puncture (LP) was conducted in 9 of 12 patients (75%), with CSF analysis findings presented in Table 2. Diagnosis of JCV infection was confirmed through detection of JCV-specific antibodies in the CSF of 8 patients (66.7%), while the remaining 4 patients (33.3%) were diagnosed via serum antibody testing. All patients showed clinical improvement, except for 1 who experienced a more prolonged recovery. Importantly, only 1 patient continued to experience long-term neurological sequala in form of “brain fog” 3 months after diagnosis based on self-reported symptoms at follow-up; official neurocognitive testing was not done.

**Table 1.** Demographics, Symptoms, and Laboratory Findings of 12 Patients Hospitalized for JCV Infection in the Mayo Clinic Health System, Northwest Wisconsin, USA

Category	%	No.
Neuroinvasive disease	75	9/12
Meningitis	42	5/12
Encephalitis	16.5	2/12
Meningoencephalitis	16.5	2/12
Outdoor activity	100	12/12
Immunosuppression	0	0/12
Dog owners	75	9/12
Symptoms		
Constitutional symptoms	100	12/12
Myalgia	83.3	10/12
Fever	75	9/12
Headache	75	9/12
Arthralgia	58.3	7/12
Chills	58.3	7/12
Rash	33.3	4/12
Sweating	33.3	4/12
Neuropathy	25	3/12
Cough and dyspnea	25	3/12
Seizure	16.7	2/12
Syncope	8.3	1/12
Laboratory findings		
Leukocytosis	58.3	7/12
Lymphocytopenia	50	6/12
Elevated transaminases	33.3	4/12
Hyponatremia	25	3/12
Brain MRI		
Brain MRI done	41.7	5/12

Abbreviations: JCV, Jamestown Canyon virus; MRI, magnetic resonance imaging.

**Table 2.** Cerebrospinal Fluid Analysis in 9 Patients who Underwent Lumbar Puncture

Patient	CSF Cell Count	CSF Protein, mg/dL	CSF Glucose, mg/dL	CSF Predominant Cells
1	14	64	69	69% lymphocytes
2	10	24	79	46% lymphocytes
3	110	72	49	92% lymphocytes
4	289	147	57	78% lymphocytes
5	5	63	59	83% lymphocytes
6	18	47	71	58% lymphocytes
7	48	49	65	86% lymphocytes
8	10	36	62	67% lymphocytes
9	20	35	86	79% monocytes

All but 1 patient had lymphocytic pleocytosis, with the exception of 1 patient in whom the predominant CSF cells were monocytes. Normal values for CSF protein are 15–45 mg/dL, for CSF glucose they are 45–80 mg/dL, and for cell count, normal is <5 cells/mm^3^.

Abbreviation: CSF, cerebrospinal fluid.

## DISCUSSION

Case series documenting the clinical characteristics of JCV infection remain scarce, with most studies reporting limited sample sizes. For example, a Canadian study conducted between 2011 and 2016 described only 5 cases of JCV infection [2]. Similarly, a study from Massachusetts, USA, covering 2013 to 2017, described 9 cases [10]. A larger investigation by Pastula et al. analyzed 31 cases across the United States between 2000 and 2013, and while this study provided valuable demographic data, it lacked a comprehensive analysis of the clinical features of the disease.

The precise factors contributing to the rise in JCV cases remain unclear; however, the increase is likely multifactorial. Like other vector-borne infections, potential contributors may include climate change, which may expand the range and activity of mosquito vectors, enhanced viral transmission, increased physician awareness, and the proliferation of white-tailed deer, an important host in the JCV transmission cycle. Between the summers of 2017 and 2019, 60 human cases of JCV were reported in Wisconsin—a significant increase compared with just 28 cases reported 5 years earlier. Epidemiological surveillance conducted in Sawyer and Washburn counties, Wisconsin, from April to August in 2018 and 2019—areas with the highest concentration of JCV cases in the United States—identified 26 mosquito species in Northern Wisconsin involved in JCV transmission. This significant increase in mosquito population, which translates to an increase in human cases, is at least partially explained by climate change [11]. Furthermore, the observed rise in cases since 2013 may partly reflect changes in serological testing protocols, particularly the inclusion of JCV in the CDC antibody panel for domestic arboviruses [6].

The age of patients in our study ranged from 19 to 75 years, with a median age of 51 years. A male predominance was observed, with men comprising 75% of the cohort, consistent with findings from previous studies [2, 9, 10]. All patients reported engaging in outdoor activities, and 75% were pet owners, specifically dog owners. This observation is noteworthy, as dog ownership may predispose individuals to more frequent outdoor activities, thereby increasing the likelihood of contracting vector-borne infections. Importantly, none of the patients reported signs of illness in their dogs. A minority of patients recalled mosquito bites. As many infected patients do not recall being bitten by an insect, patient-reported insect bite history holds limited epidemiological and clinical value. Therefore, recall of an insect bite should not be considered a reliable diagnostic criterion. Instead, diagnosis should be guided by factors such as seasonality, geographic location, clinical presentation, and laboratory findings.

Human infections with JCV exhibit a wide clinical spectrum, ranging from asymptomatic infection to nonspecific febrile illness and, in severe cases, neuroinvasive diseases such as meningitis or encephalitis. In immunocompromised individuals, JCV infections can result in fatal outcomes or prolonged hospitalizations with significant complications, particularly when coinfections with other neuroinvasive pathogens are present [7, 8]. Interestingly, in our cohort none of the 12 patients were immunocompromised. One patient who had prostate cancer was in remission and was not on active treatment. When symptomatic, JCV is typically present as acute illness and is rarely chronic or fatal. However, fatal cases have been reported in immunocompromised individuals, particularly those undergoing B-cell-depleting therapies [12].

Some illustrative cases from our cohort include the 2 pregnant patients who were in their first trimester at the time of infection. The symptoms of the disease were not different than in other nonpregnant women, and both delivered healthy infants. To the best of our knowledge, these 2 are the first reports of the outcome of JCV infection in pregnancy with no negative sequelae in either the patients or their infants.

As demonstrated in our case series and in the previously published cohort from Massachusetts, some patients may present with syncope and seizures [10]. Two patients in our cohort exhibited seizure as the sole manifestation of JCV infection, with normal neuroimaging but evidence of probable encephalitis, based on 1 major and 3 minor criteria proposed by the International Encephalitis Consortium [13]. One patient's clinical course was further complicated by rhabdomyolysis and acute kidney injury, likely secondary to the seizure rather than direct cytopathogenic effects of the virus. Myositis and rhabdomyolysis have not been reported so far to be complications of JCV infection, and in this case, prolonged seizures are more likely to be the cause of rhabdomyolysis.

Another interesting observation from our cohort was that some patients developed neuropathy as a prodrome before other symptoms developed, and in our cohort 2 patients developed neuropathic pain in the scalp area and 1 in the bilateral lower extremities. These symptoms were the first to occur, and all recovered within 2 weeks of diagnosis. Back pain is another manifestation that 50% of patients reported, which in similar fashion resolved within 2 weeks of onset.

This case series underscores the diagnostic challenges associated with the diagnosis of JCV infection. Clinically, JCV infection shares significant overlap with other neuroinvasive arboviral infections and can mimic endemic tick-borne diseases such as neuroborreliosis and Powassan virus encephalitis. Brain MRI findings are often normal or nonspecific, with findings of leptomeningeal enhancement offering limited diagnostic utility, and no pathognomonic MRI features are currently recognized for neuroinvasive JCV infection. CSF analysis frequently reveals lymphocytic pleocytosis, a nonspecific finding also reported in other case series, further complicating the differentiation between JCV and other neuroinvasive arboviral infections. Clinical differentiation among diseases caused by members of the California serogroup—including La Crosse virus, Snowshoe Hare virus, Tahyna virus, Jamestown Canyon virus, and Inkoo virus—remains challenging due to overlapping clinical features and significant serological cross-reactivity. This cross-reactivity not only complicates clinical diagnosis but also poses considerable challenges in laboratory confirmation, particularly when relying on serologic testing alone [14, 15].

La Crosse virus is the most common cause of severe encephalitis in children, whereas JCV more frequently affects adults. Infection with La Crosse virus is often associated with more severe neurological manifestations, including seizures and focal neurological deficits. In contrast, JCV, while generally presenting with milder symptoms, can also cause seizures, encephalopathy, and peripheral neuropathy, as demonstrated in our case series [14, 15].

Snowshoe Hare virus closely resembles La Crosse virus in its clinical presentation but typically causes milder symptoms and is a significantly rarer cause of meningoencephalitis. Tahyna virus infection less commonly results in severe neurological deficits and typically presents with nonspecific symptoms such as fever, headache, and myalgia, usually in the absence of meningitis or encephalitis. Inkoo virus is rarely associated with human disease [14, 15].

Seropositivity rates for JCV among adults can approach 30% in certain geographic areas, such as Michigan [16], which may contribute to diagnostic uncertainty. For example, a case involving a patient from New Jersey initially diagnosed with Powassan virus encephalitis also was positive for JCV antibodies [4]. Subsequent clinical evaluation and testing of convalescent sera indicated Powassan virus as the likely causative agent, underscoring the challenges in diagnosing arboviral infections and the complexities in interpreting laboratory results. These findings emphasize the importance of clinical context and epidemiological factors in the accurate diagnosis of JCV and similar infections. Furthermore, in patients receiving B-cell-depleting therapies, standard serological testing may yield negative results, highlighting the limitations of antibody-based diagnostics in this population [7].

In our case series, all patients achieved full recovery. Fatal outcomes are rare and have generally been documented only in patients with coinfections involving other neuroinvasive pathogens (eg, varicella-zoster virus or Powassan virus) or in those with significant immunosuppression [7, 8, 11]. Despite recovery, neuropsychological sequelae may persist for prolonged periods, with symptoms such as mental fog potentially lingering for up to 10 weeks. Fatigue may also remain unresolved in some patients, and in rare cases instances, patients may experience persistent functional impairments, including an inability to return to work [10].

The strength of this case series lies in its comprehensive analysis of clinical presentations and patient outcomes. Notably, it is the first to report outcomes of this infection during the first trimester of pregnancy. Although it includes only 12 patients, it represents one of the largest case series currently available in the literature. The primary limitations include the absence of epidemiological data regarding the incidence of infection and the proportion of positive diagnostic tests relative to the total number of patients evaluated for encephalitis and meningitis.
